# Beyond the respiratory tract: a case of acute viral myositis following respiratory syncytial virus infection

**DOI:** 10.3205/dgkh000658

**Published:** 2026-06-23

**Authors:** Pelagia Kefala-Karli, Leandros Sassis, Aristos Aristodimou, Loukia Dramiotou, Andreas Kostis

**Affiliations:** 1 Internal Medicine Department, Limassol General Hospital, State Health Organization Services, Limassol, Cyprus

**Keywords:** acute myositis, respiratory syncytial virus (RSV), adult, atypical presentation, muscle weakness

## Abstract

**Aim:**

Acute viral myositis is an uncommon complication that typically occurs during the recovery phase of viral infections and is most frequently observed in children. It is rarely reported in adults and usually presents as myalgia of the proximal upper and lower limbs. This case report describes an atypical presentation of acute myositis in an immunocompetent adult occurring shortly after RSV infection.

**Methods:**

A 68-year-old male patient presented with sudden-onset bilateral lower limb myalgia and weakness following recent RSV infection. Laboratory evaluation revealed elevated C-reactive protein with normal creatine kinase levels. The extensive infectious and autoimmune workup was not revealing. MRI, muscle biopsy, and electrophysiological studies were conducted to further investigate the etiology of the symptoms.

**Results:**

MRI demonstrated diffuse bilateral muscle edema consistent with inflammatory myositis, while muscle biopsy and electrophysiological studies were unremarkable. The patient received supportive care, including hydration, analgesia, and rest, without corticosteroids or antivirals. Complete recovery occurred within two months.

**Conclusion:**

This case highlights an atypical presentation of RSV-associated myositis in an adult patient characterized by normal creatine kinase levels but elevated inflammatory markers. Increased awareness of this rare entity may facilitate prompt diagnosis, and prevent unnecessary diagnostic investigations as well as inappropriate immunosuppressive therapy.

## Zusammenfassung

**Zielsetzung:**

Die akute virale Myositis ist eine seltene Komplikation, die typischerweise in der Rekonvaleszenzphase viraler Infektionen auftritt und am häufigsten bei Kindern beobachtet wird. Bei Erwachsenen wird sie nur selten berichtet und manifestiert sich meist als Myalgie der proximalen oberen und unteren Extremitäten. Der Fallbericht beschreibt die atypische Präsentation einer akuten Myositis bei einem immunkompetenten Erwachsenen kurz nach einer RSV-Infektion.

**Methode:**

Ein 68-jähriger Mann stellte sich mit plötzlich aufgetretenen bilateralen Myalgien der unteren Extremitäten und Muskelschwäche nach kürzlich durchgemachter RSV-Infektion vor. Die Laboruntersuchung zeigte erhöhtes C-reaktives Protein bei normalen Kreatinkinasewerten. Die umfassende infektiologische und autoimmune Abklärung blieb ohne richtungsweisenden Befund. Zur weiteren Abklärung wurden eine Magnetresonanztomographie (MRT), eine Muskelbiopsie sowie elektrophysiologische Untersuchungen durchgeführt.

**Ergebnisse:**

Das MRT zeigte ein diffuses bilaterales Muskelödem als Ausdruck einer entzündlichen Myositis, während Muskelbiopsie und elektrophysiologische Untersuchungen unauffällig waren. Der Patient wurde konservativ mit Flüssigkeitssubstitution, Analgesie und Ruhe ohne Verabreichung von Kortikosteroiden und ohne antivirale Therapie behandelt. Innerhalb von zwei Monaten kam es zur vollständigen klinischen Erholung.

**Schlussfolgerung:**

Die Kasuistik zeigt die atypische Präsentation einer RSV-assoziierten Myositis bei einem erwachsenen Patienten, gekennzeichnet durch normale Kreatinkinasewerte bei gleichzeitig erhöhten Entzündungsparametern. Ein erhöhtes Bewusstsein für dieses seltene Krankheitsbild kann zu einer schnelleren Diagnose beitragen und unnötige diagnostische Maßnahmen sowie eine unangemessene immunsuppressive Therapie vermeiden.

## Introduction

Acute viral myositis is an uncommon inflammatory muscle disorder, typically presenting with sudden onset of generalized muscle weakness, pain, swelling, and tenderness during the convalescence stage of infection. It is most frequently associated with influenza A and B viruses, predominantly affecting the pediatric population [1]. Adult cases are rare and often present a diagnostic challenge due to clinical overlap with other neuromuscular, inflammatory, or systemic conditions [2].

Respiratory syncytial virus (RSV) is a well-described cause of upper and lower respiratory tract infections, particularly in infants, the elderly, and immunocompromised individuals. Nowadays, RSV has gained increasing recognition as a clinically significant pathogen in adults, contributing to substantial morbidity and hospitalizations [3]. Nevertheless, RSV has only rarely been implicated as a cause of acute myositis or rhabdomyolysis, especially in immunocompetent adults [4].

The diagnosis of viral myositis is primarily clinical and is supported by elevated muscle enzymes in the context of a recent viral illness, with exclusion of alternative causes [5]. Given the rarity of RSV-associated myositis in adults, awareness of this entity is limited. Here we report a rare case of acute myositis following RSV infection in an immunocompetent adult with normal muscle enzymes, aiming to expand the existing literature and highlight the importance of considering RSV in the differential diagnosis of acute myositis in adults. To our knowledge, reports of RSV-associated myositis with normal CK levels in immunocompetent adults remain extremely limited.

## Case presentation

A 68-year-old male presented to the emergency department with a 10-day history of acute onset severe bilateral proximal lower limb myalgia and progressive proximal muscle weakness, resulting in impaired ambulation. He reported intermittent episodes of rigors, although no fever was recorded. His medical history was remarkable for a recent RSV infection confirmed by a positive respiratory antigen test approximately 15 days prior to presentation. His past medical history also included diabetes mellitus, arterial hypertension, benign prostatic hyperplasia, and dyslipidemia. The patient denied any prior history of neuromuscular disease or exercise intolerance in the past.

Neurological examination revealed diffuse muscle tenderness and reduced muscle strength in the proximal lower limbs (Medical Research Council – MRC grade 4/5). Deep tendon reflexes were preserved, and sensory examination was unremarkable. Gait was impaired due to weakness and bilateral ankle edema. 

Laboratory investigations on admission demonstrated normal serum creatine kinase (CK) levels (32 U/L; reference value (RV) 39–308 U/L) which remained within normal limits on serial measurements. Renal function and serum electrolytes were normal. Inflammatory markers were markedly elevated, with C-reactive protein (CRP) level of 460 mg/L (RV <5 mg/L). 

Given the markedly elevated inflammatory markers and systemic symptoms, severe bacterial infection, including occult sepsis or infectious myositis, was initially suspected. The patient was therefore empirically started on intravenous piperacillin-tazobactam and vancomycin after obtaining blood and urine cultures. 

An extensive diagnostic evaluation was undertaken. Polymerase chain reaction testing for RSV was negative; however, this assay was performed 15 days after the onset of flu-like symptoms. Notably, the patient had previously tested positive for RSV by a respiratory antigen test, and several household members were confirmed as RSV-positive during the same period, suggesting a household transmission cluster. Acute and convalescent serological testing for Epstein-Barr virus, cytomegalovirus, human immunodeficiency virus, hepatitis B and C, *Brucella* spp., *Toxoplasma gondii*, *Rickettsia typhi*, influenza virus, and SARS-CoV-2 was negative. Screening IgM antibodies for *Coxiella burnetii* were initially positive; however, this finding was not confirmed by immunofluorescence assay. As the RSV PCR test was obtained relatively late in the disease course, a false-negative result due to reduced viral shedding cannot be excluded. Therefore, RSV-associated myositis was considered the most likely diagnosis based on the documented recent RSV infection, the epidemiological link with infected household members, temporal association with the preceding respiratory illness, exclusion of alternative infectious and autoimmune etiologies, supportive MRI findings, and spontaneous clinical recovery without immunosuppressive therapy.

In the setting of normocytic anemia [red blood cell count 3.66 ×10^6^/µL (RV 4.50–5.60x10^6^/µL), hemoglobin 11.0 g/dL (RV 13.0–17.2 g/dL), hematocrit 31.2% (RV 40.0–50.0%), mean corpuscular volume 85.2 fL (RV 83.0–98.0 fL)], a peripheral blood smear was performed and demonstrated mature granulocytes without dysplastic features. 

Given the considerably elevated inflammatory markers and unexplained anemia, an extensive investigation was performed to exclude occult infection, malignancy, or systemic inflammatory disease. Subsequent bone marrow aspiration revealed no abnormal findings, and cultures of blood, urine, and bone-marrow aspirate showed no microbial growth. Autoimmune testing, including antinuclear antibodies and myositis-specific antibodies, was negative.

Computed tomography (CT) of the chest, abdomen, and pelvis demonstrated no acute pathology, apart from a 0.5 cm “tree-in-bud” nodule in the right upper lobe of the lung. Positron emission tomography (PET) imaging revealed supra- and infradiaphragmatic lymph node enlargement with discrete to moderate fluorodeoxyglucose uptake, a non-specific finding. Additional investigation with a Mantoux test and bronchoalveolar lavage yielded negative results.

Due to the persistent muscle pain, edema, and weakness, magnetic resonance imaging (MRI) of the lower limbs was performed (Figure 1 (Fig. 1)). MRI demonstrated diffuse bilateral muscle edema with hyperintense signals, consistent with acute inflammatory myositis. There was no evidence of abscess formation, necrosis, or compartment syndrome. Muscle biopsy revealed no significant histopathological abnormalities. Nerve conduction studies and needle electromyography (proximal, distal, and paraspinal muscles) were normal.

In the absence of microbiological evidence of bacterial infection and with progressive clinical improvement, antibiotic therapy was discontinued. The patient was managed conservatively with intravenous hydration, analgesia, and rest. Corticosteroids or antiviral therapy were not administered. CRP levels gradually normalized, and muscle strength improved over the following four weeks. Complete clinical and biochemical recovery was achieved within two months, with no recurrence at follow-up. 

Based on the documented recent RSV infection confirmed by a positive respiratory antigen test, the presence of RSV-positive household contacts, imaging findings consistent with inflammatory myositis, normal CK levels, and spontaneous recovery without immunosuppressive therapy, a diagnosis of acute viral myositis most likely associated with RSV infection was established.

## Discussion

Viral myositis is an uncommon, self-limited myopathy predominately affecting children, with a male predominance, and an estimated incidence of 0.23–2.6 cases per 100,000 individuals [6]. It most frequently occurs following influenza infection and is characterized clinically by acute onset of muscle pain and difficulty walking [7]. Laboratory evaluation typically reveals markedly elevated serum CK levels, reflecting skeletal muscle injury. The condition is usually benign, resolving within weeks, although monitoring is warranted to exclude complications such as rhabdomyolysis [8], [9], [10], [11]. 

Acute myositis following respiratory viral infection in adults remains an uncommon and poorly characterized complication. While elevated serum CK is traditionally regarded as a hallmark of muscle injury, this biochemical pattern is not universally observed, particularly when inflammatory processes predominate in the absence of frank myofiber necrosis [12], [13]. Recent studies have shown that post-infection myositis can present with significant clinical and imaging evidence of muscle inflammation, despite normal CK levels [14], [15], [16] . In the present case, markedly elevated inflammatory markers accompanied by normal CK levels represent an atypical manifestation of muscle involvement, underscoring limitations of CK as a sole diagnostic biomarker in post-viral myositis. Chilakuluri et al. [17] also described a case of idiopathic inflammatory myopathy in an elderly patient in whom significant muscle symptoms and elevated inflammatory markers occurred despite normal CK levels, highlighting that normal CK values do not exclude clinically significant inflammatory muscle disease.

RSV is classically associated with lower respiratory tract disease, particularly in children and older adults, and can rarely trigger extrapulmonary inflammatory syndromes, which remain poorly recognized. Infection with RSV can elicit a robust innate and adaptive immune response, including activation of macrophages, the release of pro-inflammatory cytokines such as interleukin-6 (IL-6), and widespread immune signaling that may extend to skeletal muscle tissue [18]. This inflammatory milieu can lead to muscle pain, weakness, and MRI-detectable edema even in the absence of significant muscle enzyme release, indicating an immune-driven myopathic phenotype [16]. Recent case series of post-infectious myositis in adults describe similar clinical phenotypes—proximal muscle weakness with normal or low CK levels but elevated inflammatory markers including CRP and IL-6—suggesting a phenotype of predominantly non-necrotizing inflammatory myopathy following viral respiratory infections [16]. 

The diagnostic sensitivity of CK for detecting all forms of inflammatory muscle disease has important limitations. In classic idiopathic inflammatory myopathies, CK is often elevated; however, normal CK levels do not exclude a myositis diagnosis, particularly when inflammation is localized to interstitial, perivascular, or fascial compartments rather than causing widespread myofiber necrosis [17], [19]. In these settings, CRP or other acute-phase reactants such as IL-6 may more accurately reflect systemic immune activation than CK itself. MRI, with fluid-sensitive sequences, is increasingly recognized as crucial in detected muscle edema indicative of early inflammatory changes, often preceding definitive histopathological abnormalities on muscle biopsy [20]. 

In our patient, MRI demonstrated diffuse bilateral muscle edema without features of necrosis, further supporting a non-destructive inflammatory mechanism. Muscle biopsy was non-specific, which is consistent with reports that histopathology may be normal or show only subtle inflammatory changes early in post-viral myopathies; our case likely reflects a similarly early stage [21]. Clinically, the temporal association with a recently documented RSV infection, absence of alternative infectious or autoimmune etiologies, and spontaneous recovery without immunosuppressive therapy strongly favor a diagnosis of RSV-associated inflammatory myositis rather than a primary autoimmune myopathy, which typically requires immunomodulation and often follows a relapsing course.

Early identification of post-viral inflammatory myositis using a combination of clinical history, imaging modalities, autoimmune and inflammatory biomarkers can help differentiate this condition from bacterial sepsis or autoimmune myopathies. Prospective studies are needed to better define its natural course, identify reliable biomarkers for early detection and to investigate targeted therapeutic intervention, such as cytokine-modulating therapies in cases with severe systemic inflammatory activation [16], [22]. Although most cases resolve with conservative management, severe complications such as rhabdomyolysis requiring immunosuppressive therapy have been described in the literature [23]. Recent studies highlight that, while conventional immunosuppressive therapies remain cornerstone of treatment [16], several emerging biological agents, including B-cell depletion therapies, Janus kinase inhibitors, and interleukin-targeted agents, show promising clinical benefits, particularly in refractory or severe cases [24], [25]. 

This case highlights an important clinical challenge: markedly elevated inflammatory markers in the setting of viral myositis may mimic severe bacterial infection, leading to empiric broad-spectrum antibiotic use. While early empirical therapy may be justified in the context of sepsis risk, clinicians should re-evaluate antimicrobial necessity once bacterial infection has been reasonably excluded, aligning practice with antimicrobial stewardship principles. 

## Conclusion

This case illustrates that RSV infection can lead to acute inflammatory myositis in adults, presenting with markedly elevated C-reactive protein levels, normal creatine kinase levels, and negative microbiological cultures. Recognition of this atypical presentation may help clinicians avoid unnecessary antibiotic therapy, invasive diagnostic procedures, and inappropriate immunosuppressive treatment.

## Notes

### Authors’ ORCIDs

- Kefala-Karli P: https://orcid.org/0000-0003-4265-9033
- Sassis L: https://orcid.org/0000-0003-3064-1434
- Aristodimou A: https://orcid.org/0009-0003-6567-1999
- Dramiotou L: https://orcid.org/0009-0008-8826-6730
- Kostis A: https://orcid.org/0009-0001-8172-8356

### Funding

None. 

### Competing interests

The authors declare that they have no competing interests.

## Figures and Tables

**Figure 1:**
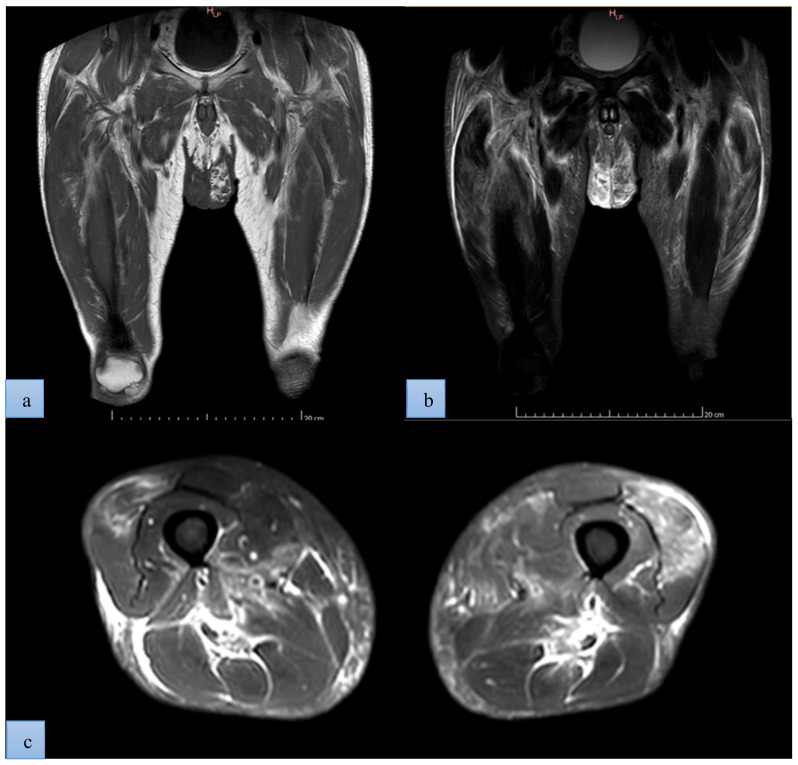
Magnetic resonance imaging with (a) coronal view of T1 sequence showing preserved muscle architecture, (b) coronal view of STIR (Short Tau Inversion Recovery) sequence demonstrating bilateral muscle edema with hyperintense signal within the affected muscles, consistent with acute inflammatory myositis and (c) transverse view of SPAIR (Spectral Adiabatic Inversion Recovery) sequence showing diffuse hyperintense signal within the affected muscles, consistent with intramuscular edema and active inflammation.
